# Genetic Diversity and Genetic Relationships of Purple Willow (*Salix purpurea* L.) from Natural Locations

**DOI:** 10.3390/ijms19010105

**Published:** 2017-12-30

**Authors:** Paweł Sulima, Kathleen Prinz, Jerzy A. Przyborowski

**Affiliations:** 1 Department of Plant Breeding and Seed Production, University of Warmia and Mazury in Olsztyn, 10-724 Olsztyn, Poland; jerzy.przyborowski@uwm.edu.pl; 2 Institute for Systematic Botany, Friedrich-Schiller-Universität Jena, 07743 Jena, Germany; kathleen.prinz@uni-jena.de

**Keywords:** *Salix purpurea* L., genetic diversity, genetic relationships, molecular markers, AFLP, RAPD, ISSR, breeding

## Abstract

In this study, the genetic diversity and structure of 13 natural locations of *Salix purpurea* were determined with the use of AFLP (amplified length polymorphism), RAPD (randomly amplified polymorphic DNA) and ISSR (inter-simple sequence repeats). The genetic relationships between 91 examined *S. purpurea* genotypes were evaluated by analyses of molecular variance (AMOVA), principal coordinates analyses (PCoA) and UPGMA (unweighted pair group method with arithmetic mean) dendrograms for both single marker types and a combination of all marker systems. The locations were assigned to distinct regions and the analysis of AMOVA (analysis of molecular variance) revealed a high genetic diversity within locations. The genetic diversity between both regions and locations was relatively low, but typical for many woody plant species. The results noted for the analyzed marker types were generally comparable with few differences in the genetic relationships among *S. purpurea* locations. A combination of several marker systems could thus be ideally suited to understand genetic diversity patterns of the species. This study makes the first attempt to broaden our knowledge of the genetic parameters of the purple willow (*S. purpurea*) from natural location for research and several applications, inter alia breeding purposes.

## 1. Introduction

Recent years have witnessed the growing popularity of natural remedies, which increased the demand for high-quality herbal material [1,2]. Willow bark is commonly used in the production of natural alternatives to aspirin because of similar medicinal properties to acetylsalicylic acid (synthetic aspirin): it delivers analgesic, antipyretic, anti-inflammatory and anti-rheumatic effects [3,4,5], and it is used as a cold remedy and an alternative treatment for rheumatic diseases [6,7,8,9].

The medicinal properties of willow bark can be attributed mainly to salicylic glycosides (SGs) whose content and composition vary significantly in bark tissue among genotypes [10]. Considerable differences in the content of pharmacologically active ingredients are observed both between and within species of the genus *Salix* [11,12]. The genus *Salix* comprises more than 400 identified species in highly diverse natural habitats [13,14], which points to an abundance of natural resources for the production of herbal therapies. The most stable and safe source of high-quality herbal material are identified or bred varieties, which can be grown under field conditions [15]. Highly suitable *Salix* spp. genotypes with a high content of SGs can be found in natural habitats and included in breeding programs as parental genotypes to obtain as high as possible effect of transgression. Purple willow (*Salix purpurea* L.) is characterized by one of the highest SG concentrations in the genus *Salix*. According to the literature, the SG content of purple willow bark ranges from 3% to 11% [10,11,12,16]. Such high variations in the SG content of *S. purpurea* L. justify breeding efforts to obtain varieties that are most suited for medicinal use. 

Breeding of new plant varieties is a long and laborious process that does not always lead to anticipated results. The selection of the most appropriate starting breeding material is thus a key determinant of breeding success. Starting materials for breeding should be selected based on a detailed analysis of genetic diversity of plant material in natural localities to provide potential sources of parental forms. The selection of parental genotypes should guarantee that the offspring will be characterized by considerable variations in value of trait. For this purpose, various *S. purpurea* genotypes should be collected and subjected to genetic diversity analysis to select the optimal starting parental forms. This goal can be accomplished with the use of DNA markers, which are the main diagnostic tools in modern plant breeding. DNA markers are valuable tools for *S. purpurea* studies, inter alia to identify species of *Salix* [17,18,19], genotypes of *S. purpurea* [20,21] and their genetic diversity [20,22,23]. They facilitate and support structural analyses of genomes, genetic mapping, identification of quantitative trait loci (QTL), marker-assisted selection (MAS) and sequencing of *S. purpurea* [24,25,26]. Nowadays, numerous marker systems are available and are generating from several hundred (RAPDs, ISSRs) to thousands of markers (DArTs—diversity arrays technology markers, SNPs—single nucleotide polymorphism markers). The optimal marker system, depending on the goal of its application, should be selected based on its efficiency as well as the time and cost of the generation method. The recently observed significant progress in research on the *Salix purpurea* genome, including the public availability of genomic sequence data [24,26], has made purple willow a model species in research on the genetic improvement of willows. This fact also allows the use of very efficient markers in these studies. The older marker systems begin to give way to methods based on the whole genomes sequencing. However, anonymous and neutral well-established marker systems such as AFLP (amplified length polymorphism), RAPD (randomly amplified polymorphic DNA) and ISSR (inter-simple sequence repeats) are suitable to characterize the basic genetic diversity of plant populations, including starting materials for breeding [27,28,29].

The search for valuable breeding material of *S. purpurea* can begin in natural localities because the purple willow is a common species in many countries [14,30,31]. Purple willow is native to Europe and Asia, but plants are also often anthropogenically introduced in large areas (inter alia North America and Europe) primarily to reduce erosion along stream banks and lake shores, for cultivation on arable land and for use in basketry [13,14,32]. The species is dioecious and outcrossing. The flowers are pollinated by wind and insects, and blooming starts before leaf development (March–April). Seeds are tiny capsules which are dispersed by wind. Three subspecies have been identified, and hybridization between *S. purpurea* and several other *Salix* species is common [13,14,30,31]. In our study area, some species such as *S. viminalis*, *S. triandra*, *S. caprea* and *S. fragilis* co-occur with *S. purpurea*, and hybridization among species may exist.

In this study, we used three different marker systems; namely, AFLP [33], RAPD [34], and ISSR [35] to analyze the genetic diversity within and between natural *S. purpurea* locations and to understand the genetic diversity patterns of the species.

## 2. Results and Discussion

### 2.1. Efficiency of the Used Marker Systems

The efficiency of the applied marker systems was evaluated based on data matrices for each marker system and a combination of all systems. This approach was adopted to compare the results in view of their economic and scientific applicability for breeding *S. purpurea*. The genetic diversity of *S. purpurea* was analyzed with two combinations of AFLP primers, 62 RAPD primers and 20 ISSR primers, which generated 159, 574 and 221 products, respectively (Table 1). The AFLP procedure is complex, but a relatively high number of polymorphic and reproducible characters can be expected per analysis after restriction, ligation, preamplification and selective amplification. Although lower reproducibility was assumed for RAPD marker system, their significance can be increased by applying the relevant procedures [36]. We therefore repeated the genotyping by RAPDs and ISSRs as well for both biological replicates of every sample, and ambiguous products were excluded from analysis. RAPDs and ISSRs revealed a lower number of scored products per PCR, but these methods are very simple based on only one PCR. Thus, the number of PCRs can be increased with comparatively little effort. In fact, the RAPD primers yielded around nine times fewer products per primer (9.3 products) than one combination of AFLP primers, whereas one ISSR primer produced a higher number of scored products (11.1) than RAPDs, but its yield was seven times lower than that of AFLPs (Table 1). In our study, an average of 711.7 polymorphic and 3.6 private products were identified for the analyzed locations (Table 2). Based on these two parameters, we observed minor differences in the efficiency of the used marker systems. Among all identified AFLP products, an average of 71% polymorphic products were scored for the tested locations, which was somewhat below the values noted for RAPDs and ISSRs (75%). In contrast, the average percentage of private products (8%) was higher for AFLPs than for ISSRs (6%) and RAPDs (4%). The observed differences in technical effort and yield, but also in expected significance of results indicate for the reasonable combination of several marker systems to provide the best approach for elucidating genetic diversity and genetic relationships of *S. purpurea* locations.

The percentage of polymorphic amplification products (*%p*), unbiased expected heterozygosities (*uHe*) and the values of Shannon diversity indices (*I*) were higher for RAPDs and ISSRs than for AFLPs, and ISSRs which generated higher values of average genetic differentiation between locations (*Φ_ST_*) than other methods (Table 1). The marker systems associated with highly polymorphic microsatellite regions such as ISSR are expected to reveal higher levels of genetic diversity [37] than RAPD and AFLP polymorphisms which are randomly distributed in the genome [38]. However, AFLPs revealed the highest scored products ratio, which usually lead to the highest efficiency of polymorphisms detection and highest discriminative power. Diversity levels of applied marker systems are dependent from variability of the target regions for primers and/or restriction enzymes, and they differ among species requiring a careful selection of the applied RAPD primer and AFLP primer combinations [37,39]. Despite some differences, our combined application of the three marker systems with different diversity natures could support the identification of complex diversity patterns within and among locations and regions, and it could be suitable and applicable i.e., in molecular breeding issues of *S. purpurea*. The overall applicability of various types of molecular markers in determinations of species identity, genetic diversity, taxonomic and phylogenetic analyses of the genus *Salix* has been described by several authors [17,18,19,21,40,41,42,43,44,45,46,47,48]. For example, Alsos et al. [49] demonstrated that AFLP marker system were highly effective in allocating 41 natural locations of *S. herbacea* to five groups corresponding to their geographic regions. Van Puyvelde and Triest [41] used ISSR marker system to evaluate the spatial isolation of *S. alba* at the level of individual plants as well as populations. The applicability of natural willow populations in breeding are supported by the studies of Trybush et al. [45] and Berlin et al. [50]. Trybush et al. [45] reported a high level of genetic diversity in 84 *S. viminalis* genotypes from natural localities, which validated the choice of breeding materials from the group of the examined genotypes. The cited authors used 38 SSR primers generating an average of seven alleles each. Interesting results were also reported by Berlin et al. [50] who relied on 38 SSR loci to analyze the genetic diversity of *S. viminalis* in natural habitats in Great Britain and Sweden, and concluded for high breeding potential due to the observed high genetic diversity. However, genetic diversity was lower in Sweden and many genotypes were genetically identical; therefore, the observed results were attributed to artificial introduction of the species to Sweden [50]. With regard to *S. purpurea*, our study confirms the high usefulness of AFLPs, RAPDs and ISSRs in genetic diversity analyses of natural locations [20,22,23,43].

### 2.2. Genetic Diversity and Analysis of Molecular Variance (AMOVA)

In our study, the genetic diversity of *S. purpurea* (*uHe* = 0.179; Table 1) was high despite lower than reported by other authors, but those analyses either relied on different marker systems [43] or did not include natural locations of *S. purpurea* [20,22,23]. Genetic differentiation (*Φ_ST_* = 0.212; Table 1) was also slightly lower than expected for plant species with comparable life-history traits (long-lived perennials, outbreeding, wind dispersal: *Φ_ST_* ≈ 0.25) [51]. Natural occurrence of *S. purpurea* was usually irregular, and the number of individuals in the sampled locations ranged from several to more than a dozen with a corresponding impact on genetic diversity and structure. Low abundance of *S. purpurea* is primarily related to the propagation of the species. Although many seeds are produced, establishment of plants is low due to low seed germination ability and viability. In addition, *S. purpurea* often form interspecific hybrids which additionally decrease the occurrence of homogeneous species in natural populations. In the analyzed locations, the genetic diversity of *S. purpurea* was similar when calculated with the use of single marker system (*uHe* = 0.164–0.187) and a combination of all systems (*uHe* = 0.179; Table 3). Genetic diversity in OL3 (Olsztyn Lakeland) was significantly lower than in the remaining locations (*I* = 0.134; *uHe* = 0.110; Table 3), but only three genotypes were analyzed. In most cases, natural locations from the same regions were more similar within than among regions (Table 4). However, higher genetic similarity among than within regions was also observed for locations of Olsztyn Lakeland (OL).

The components of genetic diversity of *S. purpurea* were determined by AMOVA. In a combined analysis of all marker types, AMOVA revealed 79% genetic variation within locations, 11% between locations and 10% between regions (Table 5). The distribution of genetic variation was similar for single marker systems (77–84% genetic variation within locations; data not shown). The results of this study confirm the general observation that most tree and shrub species are characterized by high genetic variations within natural populations [52]. In the work of Alsos et al. [49], genetic variations within *S. herbacea* populations ranged from 50% to 80%, depending on the geographic identity of the analyzed natural locations. Compared to our results, similar levels of genetic variation were observed for *S. viminalis* within natural locations (>90%) [42,48].

### 2.3. Genetic Relationships

**Figure 1:**
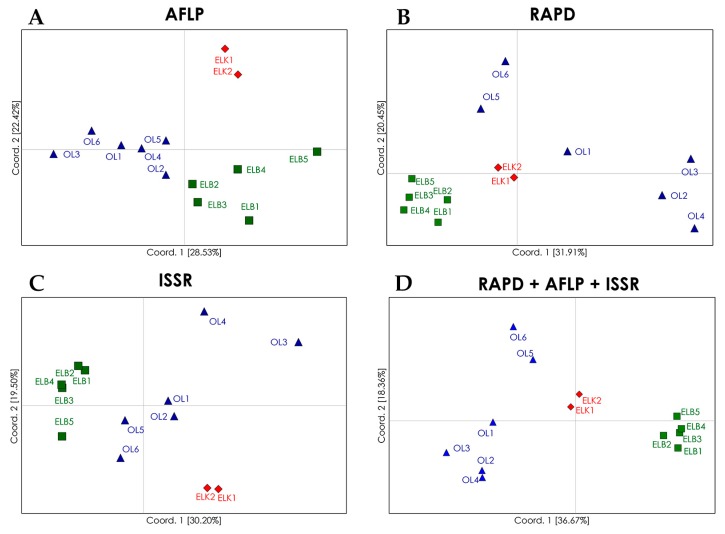
PCoA (principal coordinates analysis) plots of genetic differentiation (*Φ_ST_*) between the analyzed populations of *S. purpurea* determined with the use of: AFLP (**A**); RAPD (**B**); and ISSR (**C**) marker systems; and a combination of all marker systems (**D**). The analyzed locations are marked in different colors.

Despite low genetic variations among regions (10%; Table 5), PCoA (principal coordinates analysis) plots and dendrograms based on genetic differentiation (*Φ_ST_*) values revealed well defined clusters of *S. purpurea* locations from particular regions (Figure 1). The analyzed locations were grouped in four clusters with the use of RAPD marker system. The locations from the Ełk Lakeland (ELK) and Żuławy Wiślane (ELB) were grouped into two distinct regional clusters. The locations from region OL were separated from the clusters representing regions ELK and ELB, but they formed a non-homogeneous group of two clusters, including Cluster 3 with locations OL2, OL3 and OL4, and Cluster 4 with locations OL5 and OL6. Location OL1 was not assigned to any cluster. The allocation of genotypes from regions ELK and ELB to two separate clusters reflects their geographical distributions as both regions are separated by a considerable distance (more than 200 km): The results for region OL are surprising, because the analyzed locations are situated in relative proximity and could be expected to form a single group. In fact, this assumption is confirmed by a PCoA based on AFLP marker system, which revealed three groups corresponding to the studied regions, whereas regions OL and ELB were less distinct than region ELK. In comparison with RAPD marker system, AFLPs revealed a higher genetic similarity among locations from region OL, and lower genetic similarity among locations from region ELB. ISSR marker system showed the lowest fit between the analyzed locations and regions. Similar to AFLP and RAPD marker systems, ISSRs revealed a separate cluster of locations from region ELK. In the PCoA plot, one location from region ELB (ELB5) was clearly distinct from the remaining locations. The lowest level of genetic similarity was observed among locations from region OL, and the locations OL3 and OL4 were significantly distant from remaining locations. In contrast, in PCoA based on RAPD marker system, locations OL3 and OL4 were assigned to the same cluster. The examined locations were clearly allocated to regional groups in a combined analysis of results for the three marker systems. The locations from regions ELK and ELB formed two autonomous clusters, and locations from region OL formed two clusters, but these differences were justified. Locations OL5 and OL6 formed a separate cluster and were geographically more distant to the remaining OL locations. Despite certain differences in single marker systems, the PCoA for the combination of marker systems can be successfully used to discriminate natural locations of *S. purpurea*. The differences produced by RAPDs, AFLPs and ISSRs in this study may thus be attributed to their distribution in the genome, and thus, representation of genetic variation. The analysis based on AFLP marker system produced the best fit between the examined locations and their regions of origin, which was also reflected in the combined approach. Alsos et al. [49] also demonstrated that AFLP marker system were highly effective in allocating 41 natural locations of *S. herbacea* to five groups corresponding to their geographic regions. However, Van Puyvelde and Triest [41] relied on ISSR markers to evaluate the spatial isolation of *S. alba* at the level of individual plants as well as populations.

**Figure 2:**
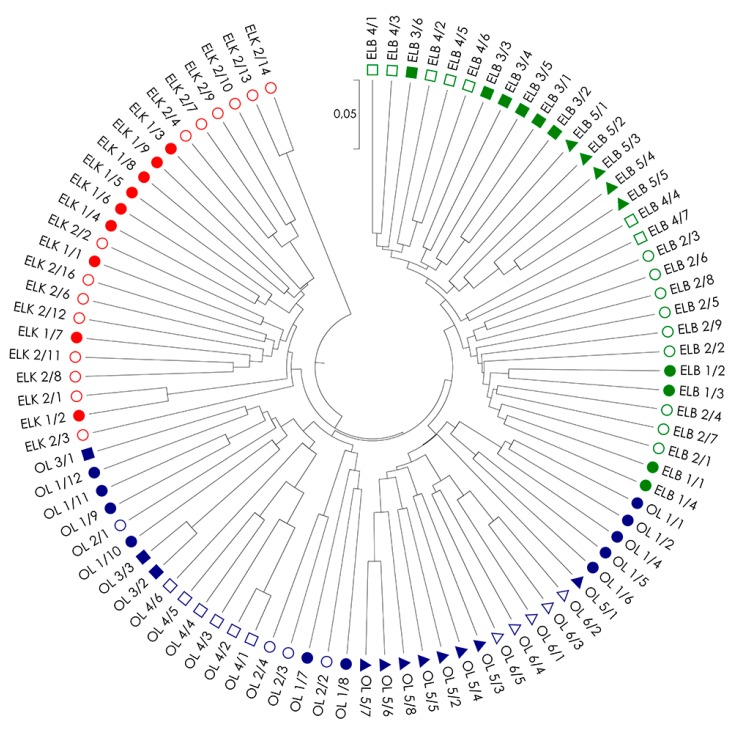
UPGMA (unweighted pair group method with arithmetic mean) dendrogram of genetic distance between 91 genotypes of *S. purpurea* based on a combined analysis of all used marker systems (AFLP, RAPD and ISSR). The analyzed locations are marked in different colors.

Genetic distances among all examined *S. purpurea* genotypes (*D_S_*; Table S1) were used to create UPGMA dendrograms for single marker systems (Figure S1) and a combination of systems (Figure 2) to illustrate the relationships among the individuals within and among locations and regions. Similar to PCoA, dendrograms also confirms the best fit between genotypes from the same regions in analysis based on a combination of all three marker types (Figure 2). The dendrogram revealed five clusters, including two clusters grouping genotypes from region ELK (with one small cluster of the two genotypes ELK 2/13 and ELK 2/14), one cluster from region ELB, and two clusters formed by genotypes from region OL. The clear separation of genotypes ELK 2/13 and ELK 2/14 from the remaining genotypes in region ELK resulted from a very low genetic distance between these genotypes (*D_S_* = 0.082) in contrast to high genetic distances among these genotypes to others in the region ELK (*D_S_* = 0.274–0.367), and among the studied genotypes in general (Table S1). The above suggests the presence of a strong kinship relations among the two genotypes. The presence of specific clusters and missing association of genotypes to their location of origin (with the exception of location OL4) could be explained by strong, but random gene flow and fragmented occurrence of the species, which often results from random establishment of the progeny.

## 3. Materials and Methods

### 3.1. Plant Material and DNA Isolation

Young leaves growing on shoots were sampled from 91 genotypes of *S. purpurea* in 13 natural locations in north-eastern Poland (Table 6) [10,53]. For our analyses, plant material was sampled only from exactly defined *S. purpurea* plants in accordance with the botanical key prepared and based on scientific literature [13,28,29,54]. The number of genotypes in every analyzed locations is related to the actual number of *S. purpurea* plants found in these locations, excluding identical genotypes (clones) identified by Sulima et al. [10]. The analyzed locations were situated in three geographic regions: Ełk Lakeland (ELK, two localities), Żuławy Wiślane—Delta of the Vistula River (ELB, five localities) and Olsztyn Lakeland (OL, six localities). DNA was isolated twice (in two biological replications) by the method proposed by Milligan with certain modifications [20,55]. The quantity and quality of DNA were evaluated with the NanoDrop 2000 spectrophotometer (Thermo Scientific, Waltham, MA, USA) and confirmed by electrophoresis.

### 3.2. AFLP Marker System

AFLP amplification products (Figure S2) were generated in accordance with the procedure described by Vos et al. [33] with certain modifications. Genomic DNA (200 ng) was digested with *Eco*RI and *Mse*I restriction enzymes (Invitrogen, Carlsbad, CA, USA), and double stranded *Eco*RI+/− and *Mse*I+/− adapters were ligated to restriction fragments. Restriction and ligation were carried out overnight at room temperature, and the obtained mixture was diluted (1:5). Preamplification was carried out using 1× Reaction buffer B (0.8 M Tris HCl pH 9.0, 0.2 M (NH_4_)_2_SO_4_, 0.2% *w*/*v* Tween-20; Solis BioDyne, Tartu, Estonia), 2.5 mM MgCl_2_, 0.2 μM dNTP, 0.2 μM of the preselective primers *Mse*I + C or G and *EcoR*I + A, 0.3 U FIREPol® DNA Polymerase (Solis BioDyne, Estonia) in 20 cycles (94 °C for 10 s, 56 °C for 30 s, 72 °C for 2 min), with a preliminary step (72 °C for 2 min) and a final step (60 °C for 30 min). In our study, 12 combinations of selective AFLP primers (Table S2) were tested on 16 randomly selected genotypes, and two of them were selected for the study generating a high number of polymorphic, repeatable and unambiguous products. The final amplification mixture was similar to the preamplification mixture, and it contained 3 μL of diluted preamplification product (1:20), 0.2 μM of *Mse*I + GAA/CTT and 0.1 μM of *EcoR*I + ACA/AAC, respectively. Amplification was carried out according to the touchdown PCR protocol in the following steps: 95 °C for 15 min, 10 cycles with an annealing ramp of 1.0 °C per cycle (94 °C for 10 s, 65–56 °C for 30 s, 72 °C for 2 min), followed by 24 cycles with annealing temperature of 56 °C, and a final polymerization at 60 °C for 30 min.

The obtained AFLP products were separated by capillary electrophoresis with the use of the ABI Prism 3100 genetic analyzer (Applied Biosystems, Foster City, CA, USA) and internal size standard GS 500 ROX™ (Applied Biosystems, Foster City, CA, USA). AFLP fragments were scored using the software Genotyper 3.7 (Applied Biosystems, Foster City, CA, USA).

### 3.3. RAPD Marker System

The total volume of PCR-RAPD reaction mixtures volume was 25 μL containing 10 ng of DNA, 1× DreamTaq™ Green Buffer (Fermentas Thermo Scientific, Waltham, MA, USA), 0.2 mM of each dNTP (Sigma Aldrich Srl, Milan, Italy), 0.4 μM of the primer and 0.625 U of DreamTaq™ Green DNA Polymerase (Fermentas Thermo Scientific, Waltham, MA, USA). The PCR reaction was performed in 37 cycles (94 °C for 30 s, 40 °C for 2 min, 72 °C for 2 min) with an initial denaturation (94 °C for 10 min) and a final elongation (72 °C for 8 min). A total of 64 RAPD primers with 10 nucleotides each (Operon Biotechnologies GmbH, Cologne, Germany) were tested, and 62 primers were used in genetic diversity analyses of *S. purpurea* generating clear and repeatable bands (Table S3). All samples were genotyped twice for both biological replications and every RAPD locus was proofed to ensure the repeatability of the results.

### 3.4. ISSR Marker System

The PCR-ISSR reaction mix (25 μL) contained 10 ng of DNA, 1× DreamTaq™ Green Buffer (Fermentas Thermo Scientific, Waltham, MA, USA), 0.2 mM of each dNTP (Sigma Aldrich Srl, Milan, Italy), 0.4 μM of the primer and 0.625 U of DNA polymerase (Fermentas Thermo Scientific, Waltham, MA, USA). The reaction was carried out in 37 cycles (94 °C for 1 min, 42–63 °C for 2 min, depending on the primer, 72 °C for 2 min) with an initial denaturation (94 °C for 1 min) and a final elongation (72 °C for 8 min). ISSR primers were selected based on literature data [25,56,57]. The annealing temperature for every primer was determined in PCR-ISSR test reactions based on a temperature gradient (Table S4). A total of 20 ISSR primers generating clear and repeatable bands were selected for the analysis. All samples were genotyped twice for both biological replications, and every ISSR locus was proofed to ensure the repeatability of the results.

The amplification products of RAPD and ISSR reactions (Figures S3 and S4) were separated on 1.5% agarose gels with TBE buffer, stained with ethidium bromide (Sigma Aldrich Chemie GmbH, Steinheim, Germany) and visualized under UV light in the DIGIDOC gel imaging system (Biogenet, Warsaw, Poland). The GeneRuler™ 100 bp DNA Ladder (100–1000 bp) (Fermentas Thermo Scientific, Waltham, MA, USA) was used as the standard.

### 3.5. Data Collection and Analysis

The results for all amplification products were transferred to a binary matrix where “1” denoted the presence and “0” denoted the absence of a product. Genetic diversity analyses were performed in GenAlEx 6.5 (The Australian National University, Canberra, Australia) [58], AFLPsurv 1.0 (Laboratory of Plant Ecology and Biogeochemistry, Université Libre de Bruxelles, Brussels, Belgium [59], Popgene 1.32 (Department of Renewable Resources, University of Alberta, Edmonton, Alberta, Canada) [60] and MEGA 7.0 (The Arizona State University, Tempe, Arizona, USA) [61]. Basic parameters were calculated to describe the effectiveness of the applied marker systems and genetic diversity within the analyzed locations of *S. purpurea*: the number and percentage of polymorphic amplification products (*pP*, *%p*), the number and percentage of private products for each location (*pM*; *%pM*), the scored products ratio (*SPR*) describing the number of scored products per primer/combination of primers, the number of detected products (*Na*) [62], unbiased expected heterozygosity (*uHe*) [63] and the Shannon diversity index (*I*) [64]. These parameters were calculated for each location based on data matrices for single marker systems and based on a combined data matrix from all marker systems. The proportion of genetic diversity components within and among locations was determined by analysis of molecular variance (AMOVA) [65]. Genetic differentiation (*Φ_ST_*) among the analyzed locations was calculated and presented in principal coordinate analyses (PCoA) [66]. A genetic distance (*D_S_*) matrix [67] was used to develop an UPGMA dendrogram [68] to illustrate the genetic relationships among the analyzed *S. purpurea* genotypes. These analyses were also performed for single marker systems and for a combination of all methods.

## 4. Conclusions

This is the first study presenting the genetic diversity of *S. purpurea* from natural localities. The observed genetic diversity is representative for willow species whose genetic structure was largely determined by the geographical regions of the studied localities. Purple willow stands are sometimes small and fragmented, which partially influences their diversity and differentiation. As expected, gene flow and migration could play an important role in wind-pollinated tree species whose small seeds are also widely dispersed by wind. This study relied on three well-established marker systems which are often used in genetic analyses of *Salix* populations. The results of single marker systems were mostly comparable, but a combined analysis of all systems is a reproducible and highly suitable method for analyzing genetic diversity and variation of *S. purpurea*. A combination of neutral genetic marker systems with different levels of genetic diversity revealed a significant discriminative power allowing for characterization and identification of genotypes and locations that constitute valuable breeding material for the pharmaceutical industry. However, the applied marker systems could be replaced in the next important breeding steps by new methods and technologies. Effective sequencing techniques reveal thousands of SNPs and contribute to the search for adaptive variation. Nevertheless, our study shows that genetic diversity and structure of natural plant locations can be effectively described with neutral, anonymous, but well-established molecular marker methods.

The presented data relating to the genetic diversity and genetic relationships between *S. purpurea* locations expand our knowledge about the biology and biogeography of the species. They indicate that analyses combining various types of DNA marker systems produce more reliable results with regard to in understand genetic diversity patterns in the species. The resulting knowledge could be valuable and useful support for breeding programs of *S. purpurea*, conducting variety surveys and construction of mapping purple willow populations.

## Figures and Tables

**Table 1:** Marker systems efficiency for analysis of genetic diversity in *S. purpurea* using AFLPs (amplified length polymorphisms), RAPDs (randomly amplified polymorphic DNA) and ISSRs (inter-simple sequence repeats) and a combination of all marker systems.

Parameter	AFLP	RAPD	ISSR	All Products
Total number of products (*L*)	159	574	221	954
Percent of polymorphic loci (*%p*)	74.8	89.9	87.3	86.8
Scored products ratio (*SPR*)	79.5	9.3	11.1	11.4
Shannon diversity index (*I*)	0.187	0.264	0.258	0.247
Unbiased expected heterozygosity (*uHe*)	0.134	0.192	0.187	0.179
Minimum *uHe* within a location (*MinuHe*)	0.096	0.114	0.091	0.110
Maximum *uHe* within a location (*MaxuHe*)	0.170	0.232	0.220	0.218
Genetic differentiation between locations (*Φ_ST_*)	0.150	0.159	0.225	0.212

**Table 2:** Polymorphic (*pP*) and private amplification products (*pM*) for the analyzed locations of *S. purpurea*.

Location	Parameter	AFLP	RAPD	ISSR	All Marker Systems
ELK1	*pP*	125	481	186	792
	*pM*	0	2	1	3
ELK2	*pP*	138	517	199	854
	*pM*	2	7	0	9
ELB1	*pP*	108	390	154	652
	*pM*	1	0	2	3
ELB2	*pP*	124	469	186	779
	*pM*	0	2	1	3
ELB3	*pP*	109	444	171	724
	*pM*	1	0	1	2
ELB4	*pP*	116	449	171	736
	*pM*	0	3	0	3
ELB5	*pP*	102	404	158	664
	*pM*	0	1	1	2
OL1	*pP*	119	492	185	796
	*pM*	4	1	1	6
OL2	*pP*	98	404	158	660
	*pM*	0	1	2	3
OL3	*pP*	95	342	125	562
	*pM*	1	1	1	3
OL4	*pP*	108	382	142	632
	*pM*	0	1	1	2
OL5	*pP*	116	456	168	740
	*pM*	2	2	0	4
OL6	*pP*	107	395	159	661
	*pM*	1	1	2	4
Average	*pP*	112.7	432.7	166.3	711.7
	*pM*	0.9	1.7	1.0	3.6
	*%p*	70.9	75.4	75.3	74.6
	*%pM*	7.5	3.8	5.9	4.9

*%p*, average percentage of polymorphic amplification products per location; *%pM*, average percentage of private products per location; ELK, Ełk Lakeland; ELB, Żuławy Wiślane; OL, Olsztyn Lakeland.

**Table 3:** Genetic diversity parameters in the studied locations of *S. purpurea* based on all DNA marker systems (RAPD, AFLP and ISSR).

Location	*N*	*Na*	*Ne*	*I*	*uHe*	*%p*
ELK1	9	1.421	1.325	0.295	0.205	59.78
ELK2	14	1.582	1.348	0.322	0.218	69.16
ELB1	4	1.057	1.250	0.212	0.164	38.04
ELB2	9	1.382	1.323	0.288	0.202	57.61
ELB3	6	1.220	1.286	0.248	0.181	46.88
ELB4	7	1.285	1.300	0.266	0.190	51.77
ELB5	5	1.082	1.261	0.220	0.165	39.40
OL1	11	1.454	1.339	0.306	0.211	62.36
OL2	4	1.090	1.270	0.228	0.178	40.76
OL3	3	0.829	1.157	0.134	0.110	23.51
OL4	6	1.056	1.249	0.212	0.157	38.99
OL5	8	1.317	1.306	0.273	0.193	53.94
OL6	5	1.067	1.245	0.209	0.156	38.45
Average	7.0	1.218	1.282	0.247	0.179	47.74

*N*, sample size; *Na*, number of different alleles; *Ne*, average number of effective alleles; *I*, Shannon diversity index; *uHe*, unbiased expected heterozygosity; *%p*, percent of polymorphic loci.

**Table 4:** Genetic differentiation *Φ_ST_* (below diagonal) between the analyzed locations of *S. purpurea* based on AFLP, RAPD and ISSR results.

Location	ELK1	ELK2	ELB1	ELB2	ELB3	ELB4	ELB5	OL1	OL2	OL3	OL4	OL5	OL6
**ELK1**	0.000												
**ELK2**	0.028	0.000											
**ELB1**	0.194	0.173	0.000										
**ELB2**	0.166	0.146	0.004	0.000									
**ELB3**	0.188	0.167	0.081	0.068	0.000								
**ELB4**	0.187	0.154	0.106	0.082	0.045	0.000							
**ELB5**	0.222	0.188	0.166	0.131	0.132	0.131	0.000						
**OL1**	0.160	0.149	0.190	0.172	0.184	0.191	0.219	0.000					
**OL2**	0.221	0.233	0.244	0.235	0.265	0.291	0.312	0.098	0.000				
**OL3**	0.244	0.224	0.322	0.257	0.319	0.318	0.369	0.101	0.222	0.000			
**OL4**	0.241	0.238	0.287	0.260	0.269	0.280	0.317	0.158	0.190	0.222	0.000		
**OL5**	0.181	0.157	0.216	0.172	0.209	0.202	0.229	0.098	0.234	0.223	0.242	0.000	
**OL6**	0.249	0.201	0.294	0.239	0.257	0.280	0.300	0.143	0.271	0.296	0.292	0.119	0.000

**Table 5:** Analysis of molecular variance (AMOVA) based on all 954 amplification products (AFLP, RAPD and ISSR) for 91 genotypes of *S. purpurea* (*p* < 0.01).

Source of Variation	Df	Sum of Squares	Mean Square	Estimated Variation	Total Variance
Between regions	2	1314.12	657.06	14.17	10%
Between locations	10	2005.46	200.55	14.46	11%
Within locations	78	8319.15	106.66	106.66	79%
Total	90	11,638.74	–	135.28	100%

**Table 6:** Geographic information relating to the analyzed natural locations of *S. purpurea*.

Location	Latitude/Longitude	Geographic Region	Number of Genotypes
ELK1	53°48′59.94″/22°23′12.06″	Ełk Lakeland—ELK	9
ELK2	53°50′23.52″/22°22′41.62″	Ełk Lakeland—ELK	14
ELB1	54°8′37.35″/19°23′4.98″	Delta of the Vistula River—ELB	4
ELB2	54°7′34.18″/19°18′28.95″	Delta of the Vistula River—ELB	9
ELB3	54°11′17.02″/19°12′29.94″	Delta of the Vistula River—ELB	6
ELB4	54°15′30.93″/19°14′18.96″	Delta of the Vistula River—ELB	7
ELB5	54°9′14.92″/19°1′10.77″	Delta of the Vistula River—ELB	5
OL1	53°45′30.02″/20°29′12.62″	Olsztyn Lakeland—OL	11
OL2	53°46′18.72″/20°26′36.12″	Olsztyn Lakeland—OL	4
OL3	53°46′43.26″/20°30′42.54″	Olsztyn Lakeland—OL	3
OL4	53°43′3.06″/20°28′18.72″	Olsztyn Lakeland—OL	6
OL5	53°52′31.56″/20°21′25.14″	Olsztyn Lakeland—OL	8
OL6	53°51′54.90″/20°22′45.30″	Olsztyn Lakeland—OL	5

## Supplementary Materials

Supplementary Materials can be found at http://www.mdpi.com/1422-0067/19/1/105/s1.
